# Impulsive and Partial Insanity

**Published:** 1886-08

**Authors:** 


### EDITORIAL.

### IMPULSIVE AND PARTIAL INSANITY.

The following rule was laid down by the trial judge in the State *v.* Nixon in the district court of Kansas : A person to be held criminally responsible must know the nature of the act, that it was wrong, *and have the will and the mental power to do or not to do it.* The supreme court comments upon this ruling, though it was not directly raised in the points on appeal. The court said, in substance, that it was inexpedient to admit that a person could commit crime of the nature of which he was fully conscious while under the influence of a morbid impulse sufficient to paralyze his will. The doctrine of "expediency" here affirmed has its prototype in many previous rulings. We doubt if the interests of society can be conserved by the punishment of those who are irresponsible. The question is one as to fact and not expediency. Can a person suffer from disease of the brain, in consequence of which he may commit acts the moral quality of which he fully understands, and which fill him with the greatest distress and abhorrence? We think those of even limited experience in the care of insane will admit the existence of a large number of this class of cases. Many acts of the insane which have little or no ethical significance belong to this category, and we must study the conduct of the insane as a whole. Such being the fact, it is the duty of the courts to admit it and to formulate rules of practice that will conserve society and protect the innocent.

The doctrine of partial insanity, as stated in the same case, that a person may be insane and irresponsible on one subject or set of subjects, and on all others be fully sane and responsible, is much more readily admitted by lawyers than by physicians. It has a certain warrant in the fact that all insanity in which there is not imbecility or complete overthrow of the mind is more or less "partial"; that is, it assumes a certain form and direction, in which the mental impairment is most pronounced. The difficulty lies in deciding regarding that "reasonable doubt" for which the law provides. Can an individual who has suffered impairment of his mental functions to such a degree as to develop delusion, be considered sane beyond a *reasonable doubt* in matters not directly connected with that delusion? We think such a view is hardly in accord with our present knowledge of the physiology and pathology of the brain.

The rule of partial insanity, or monomania as expressed by the court, is of extremely limited and doubtful application. A very few cases that might be included under this head have been reported. The writer in a somewhat extensive experience has seen but one, and that may have been imperfectly diagnosed.

